# Long Noncoding RNA LINC00909 Induces Epithelial-Mesenchymal Transition and Contributes to Osteosarcoma Tumorigenesis and Metastasis

**DOI:** 10.1155/2022/8660965

**Published:** 2022-10-10

**Authors:** Wanshun Liu, Qi Zhang, Kai Shen, Keran Li, Jie Chang, He Li, Ao Duan, Sheng Zhang, Yumin Huang

**Affiliations:** ^1^Department of Orthopedics, The First Affiliated Hospital of Nanjing Medical University, 300 Guangzhou Road Nanjing, 210029 Jiangsu, China; ^2^Department of Orthopedics, Nanjing First Hospital of Nanjing Medical University, Nanjing 210006, China; ^3^Department of Pain Management, Sir Run Run Hospital, Nanjing Medical University, Nanjing 211100, China; ^4^Department of Cataract, The Affiliated Eye Hospital of Nanjing Medical University, 138 Hanzhong Road, Nanjing, 210003 Jiangsu, China; ^5^Department of Orthopedics, Zhongda Hospital, School of Medicine, Southeast University, No. 87 Ding Jia Qiao, Nanjing, 210009 Jiangsu, China

## Abstract

**Background:**

Osteosarcoma (OS) is a malignant tumor that is highly metastatic with a high mortality rate. Although mounting evidence suggests that LINC00909 is strongly associated with the malignant progression of various tumors, the exact role of LINC00909 in OS remains unknown. Therefore, the current study was designed to investigate the relationship between LINC00909 and the malignant progression of OS.

**Methods:**

LINC00909 expression was measured in OS cell lines and clinical specimens using RT-qPCR assays. The effects of LINC00909 on OS proliferation, invasion, and migration were calculated both in vitro and in vivo. Apart from that, bioinformatics analyses, FISH, RIP, and luciferase reporter assays were carried out to investigate the downstream target of LINC00909. Rescue experiments were also conducted to investigate the potential mechanisms of action of competitive endogenous RNAs (ceRNAs).

**Results:**

In this study, we found that LINC00909 was highly expressed in OS cell lines and clinical specimens. In vivo and in vitro experiments demonstrated that LINC00909 induces epithelial-to-mesenchymal transition (EMT) and contributes to OS tumorigenesis and metastasis. FISH, RIP, and luciferase assays indicated that miR-875-5p is a direct target of LINC00909. Moreover, HOXD9 was validated as the downstream target of miR-875-5p in a luciferase reporter assay and western blotting experiments. Rescue experiments revealed that HOXD9 reversed the effect of LV-sh-LINC00909 on OS cells by positively regulating the PI3K/AKT/mTOR signaling pathway.

**Conclusion:**

Collectively, LINC00909 induces EMT and contributes to OS tumorigenesis and metastasis through the PI3K/AKT/mTOR pathway by binding to miR-875-5p to upregulate HOXD9 expression. Targeting the LINC00909/miR-875-5p/HOXD9 axis may have potential in treating OS.

## 1. Introduction

Osteosarcoma (OS) is a common primary bone tumor mostly seen in children and adolescents [1]. The treatment of primary tumors in patients with OS is mainly chemotherapy and surgery [2]. Despite great improvements in neoadjuvant chemotherapy and surgery, the possibility of long-term survival for patients with metastases is still low [3]. Therefore, it is critical to elucidate the mechanism underlying OS metastasis.

Long noncoding RNAs (lncRNA) are a class of RNA molecules greater than 200 nucleotides that are involved in various biological processes such as metastasis or tumorigenesis. lncRNAs affect these processes by modulating epigenetic, transcriptional, and posttranscriptional gene expression [4, 5]. Notably, lncRNAs may play a role as competing endogenous RNAs (ceRNAs), which bind miRNAs that target mRNA expression [6]. Moreover, lncRNAs modulate numerous OS biological processes, like epithelial-mesenchymal transition (EMT), cell growth, and apoptosis [7–9]. LINC00909 is upregulated in many tumor types and regulates tumor malignant progression through the ceRNA mechanism [10, 11]. Nevertheless, LINC00909's role in OS is still unclear.

In EMT, epithelial cells lose their polarity and tight and adhesion junctions between cells and gain infiltration and migration capabilities to become cells with mesenchymal characteristics [12, 13]. During the malignant evolution of tumors, EMT enables tumor cells to invade and metastasize [14, 15]. Therefore, inhibiting the progression of EMT may be a potentially effective treatment for OS.

MicroRNAs (miRNAs) are noncoding RNAs that contain 22–28 nt, which promote target mRNA decomposition and suppress its translation through binding to its 3′-untranslated regions (UTR) [16]. It has been discovered that miRNAs are linked to tumor occurrence and progression [17]. Downregulated miR-875-5p expression can be detected in numerous types of tumors and associated with a favorable prognosis [18, 19]. However, the function of miR-875-5p in OS progression is still unknown.

HOXD9, a transcription factor (TF), has an essential function in the HOX family, which encodes for transcription factors with crucial roles in development [20]. Studies have shown that HOXD9 is highly expressed in diverse cancers, which predicts a dismal prognostic outcome [21, 22]. In addition, HOXD9 expression is linked to tumor proliferation, invasion, and distant metastasis [23, 24]. However, the relationship between HOXD9 and osteosarcoma progression has not been reported.

The present study analyze LINC00909's function in OS progression by experiments in vitro and in vivo and explore the related mechanisms. LINC00909 functions as a ceRNA to bind miR-875-5p, thereby upregulating HOXD9 expression, and contributes to OS tumorigenesis and metastasis through the PI3K/AKT/mTOR pathway. This study illustrates a new possible mechanism of OS development and provides a rationale for a novel anti-OS therapeutic strategy.

## 2. Materials and Methods

### 2.1. Clinical Specimens

We collected 60 OS patients who received tumor biopsies prior to radiotherapy and chemotherapy in the Department of Orthopedics of Jiangsu Provincial People's Hospital between 2014 and 2020. Three pathologists confirmed the histological diagnosis of intraoperatively resected OS samples. Both tumor and matched noncarcinoma samples were frozen in liquid nitrogen. All patients provided informed consent for participation. This study gained approval from the Evaluation Committee and Ethics Committee of the First Affiliated Hospital of Nanjing Medical University. Clinicopathological features of patients are presented in Table 1.

### 2.2. Cell Culture

OS cells were cultivated within DMEM (Gibco, CA, USA) that contained 1% penicillin/streptomycin (P/S) (Gibco) as well as 10% fetal bovine serum (FBS, Gibco, NY) at 37°C, while hFOB1.19 osteoblasts were cultivated in DMEM that contained 1% P/S and 10% FBS at 33.5°C. The above cell lines were subject to incubation in 5% CO_2_.

### 2.3. Cell Transfection and Lentivirus Construction

The LINC00909 overexpression lentiviral plasmid LV-LINC00909, lentiviral plasmid containing short hairpin RNA (shRNA) targeting LINC00909, plasmid containing shRNA targeting HOXD9, HOXD9 overexpression plasmid, and the corresponding negative control plasmids were purchased from Tsingke (Nanjing, China). Additional file 1: Table S1 provides the shRNA sequences. RT-qPCR was conducted to verify transfection efficiency. LV-sh-LINC00909#3 and sh-HOXD9#1 showed the greatest knockdown efficiency, which was therefore chosen as the optimal shRNA for later studies. The miR-875-5p inhibitor and miR-875-5p mimic, together with corresponding negative controls, were provided by RioBio (Guangzhou, China). Lipofectamine 3000 (Invitrogen, CA, USA) was used to transfect OS cells according to the manufacturer's instructions.

### 2.4. Bioinformatics Analyses

The lncRNA-sequencing data of corresponding clinical information OS were downloaded from the TARGET database; R package limma was used to analyze lncRNA-sequencing data. The downstream miRNA targets of LINC00909 were predicted using the DIANA tool and the lncRNASNP2 tool. Gene microarray data (GSE12865 and GSE14359) for OS were downloaded from the Gene Expression Omnibus database. The starBase tool was used to find the targets of miR-875-5p. The lncRNASNP2 tool was used to predict the targeted relationship between miR-875-5p and LINC00909. TargetScanHuman 7.2 was used to predict the targeted relationship between miR-875-5p and HOXD9. Gene Set Enrichment Analysis (GSEA) was performed to detect functions of LINC00909 and the downstream signaling pathways of HOXD9 in OS based on the TARGET database.

### 2.5. RNA Extraction and Quantitative Real-Time PCR

TRIzol (Invitrogen, United States) was used to isolate total tissue and cell RNA. A NanoDrop spectrophotometer was utilized to measure RNA content and purity. In addition, RNeasy/miRNeasy Mini Kits (Qiagen) were employed to extract miRNAs. We conducted RT-qPCR according to previous publications [25]. U6 and *β*-actin were the reference for normalizing miRNA and LINC00909/HOXD9 levels, respectively. Every experiment was carried out in triplicate. The 2^−*ΔΔ*CT^ method was used to determine relative gene levels. Sequences of all primers utilized are displayed in Additional file 1: Table S1.

### 2.6. Western Blotting

The total proteins of tissues and OS cells were extracted by RIPA lysis buffer (YEASEN, China); then, the BCA protein detection kit (Thermo Fisher Scientific, USA) was adopted for measuring protein concentration. Samples were boiled, denatured, and separated using electrophoresis. Proteins were then transferred onto PVDF membranes. Rapid blocking solution was used to block membranes for a 30 min period. Primary antibodies were then used to incubate membranes at 4°C overnight. Afterwards, membranes were further incubated with secondary antibody for an additional 1 h at room temperature (RT). The membranes were then exposed to ECL reagent (Millipore, USA) with the Tanon 4200 automatic chemiluminescence imaging analysis system. Antibody information is shown in Additional file 2: Table S2.

### 2.7. 5-Ethynyl-2-deoxyuridine Incorporation, Colony Formation, and Cell Counting Kit-8

To measure OS cell proliferation, 5-Ethynyl-2-deoxyuridine (EdU), colony formation, and Cell Counting Kit-8 (CCK-8) assays were performed as described previously [25].

### 2.8. Scratch and Transwell Assays

Scratch and transwell assays were carried out to determine the impacts on OS cell invasion and migration according to previous experiments [25].

### 2.9. Fluorescence In Situ Hybridization

LINC00909 levels in OS, matched noncarcinoma tissues, and OS cells were detected using a fluorescence in situ hybridization (FISH) assay. Probes against LINC00909 and miR-875-5p were synthesized by Servicebio (Wuhan, China). FISH was performed according to the manufacturer's protocol. An upright fluorescence microscope (Nikon, Japan) was used for imaging.

### 2.10. RNA Isolation of Cytoplasmic and Nuclear Fractions

The cytoplasmic and nuclear fractions were extracted using the PARIS™ Kit (Thermo Fisher, MA, USA). RT-qPCR was conducted to analyze the expression levels of LINC00909, 18S (cytoplasmic control transcript), and U6 (nuclear control transcript).

### 2.11. Luciferase Reporter Assay

LINC00909's binding sites for miR-875-5p were predicted using the lncRNASNP2 tool (http://bioinfo.life.hust.edu.cn/lncRNASNP). HOXD9 binding sites for miR-875-5p were predicted using the TargetScan database (http://www.targetscan.org/vert_72/). Mutant (MUT) and wild-type (WT) LINC00909, named MUT-LINC00909-3′ UTR and WT-LINC00909-3′ UTR, respectively, together with MUT and WT HOXD9, named MUT-HOXD9-3′ UTR and WT-HOXD9-3′ UTR, respectively, were prepared using GenScript (Nanjing, China). MG63 and 143B cells were first transfected with miR-NC or miR-875-5p mimic, followed by cotransfection with MUT-LINC00909-3′ UTR, WT-LINC00909-3′ UTR, MUT-HOXD9-3′ UTR, and WT-HOXD9-3′ UTR for a 48 h period. We then utilized the double Luciferase Assay System (Promega, USA) to measure luciferase activity. Normalization was based on Renilla luciferase activity.

### 2.12. RNA Immunoprecipitation (RIP)

Magna RIP RNA-binding Protein IP Kit (Millipore, Billerica, MA) was used to perform the RIP assay. 143B and MG63 cells were lysed using RIP lysis wash buffer. After being centrifuged for half an hour, the supernatant was subjected to immunoprecipitation with anti-IgG or anti-Ago2-coated magnetic beads. RT-qPCR was used to detect RNA levels in the precipitates.

### 2.13. Immunohistochemistry

A 4% paraformaldehyde solution was used to fix nude mouse and human cancer samples; samples were then embedded in paraffin and sliced into 4 *μ*m sections for immunohistochemistry (IHC). Antigen retrieval and blocking were performed, followed by incubation with anti-vimentin and anti-Ki-67 primary antibodies at 4°C overnight. Slides were then incubated for 1 h with secondary antibodies at ambient temperature. Sections were then treated with freshly prepared 3,3-diaminobenzidine solution. The staining intensity and positive tumor percentage were measured from five randomly selected fields of view.

### 2.14. Animal Experiments

Twenty 6-week-old female athymic BALB/c nude mice were divided into four groups for xenograft experiments: LV-LINC00909, LV-sh-LINC00909, and the corresponding NC groups, *N* = 5 for each. An OS cell suspension (200 *μ*l) of 2 × 10^7^ luciferase-expressing cells/ml was injected into the anterior right armpit of each animal. We measured tumor dimensions every four days and calculated volumes according to the following formula: volume = (width)^2^ × length/2. At 28 days postcell implantation, the fluorescence intensity of tumors was detected using the IVIS Imaging System (Caliper Life Sciences, USA) and tumor tissues were resected, weighed, and fixed for IHC assays. Additionally, the OS lung metastasis model was constructed in 6-week-old female athymic BALB/c nude mice. Mice were grouped similarly to above, and 100 *μ*l of cell suspension containing 2 × 10^7^/ml of OS cells transfected with a luciferase-expressing vector was injected into each mouse through the tail vein. Twenty-eight days postinjection, we measured the fluorescence intensity of lung metastases using the IVIS imaging system and lung tissue was removed for formalin fixation and subsequent hematoxylin and eosin.

### 2.15. Statistical Analysis

Results were presented in a form of mean ± SD. All assays were carried out in triplicate. SPSS22.0 (SPSS Inc., Chicago, Illinois, USA) was employed for statistical analysis. The significance between two groups was compared using a Student *t*-test, whereas a one-way ANOVA was adopted for comparison across several groups. *p* < 0.05 was considered statistically significant.

## 3. Results

### 3.1. LINC00909 is Highly Expressed in OS Tissues and Cells

**Figure 1:**
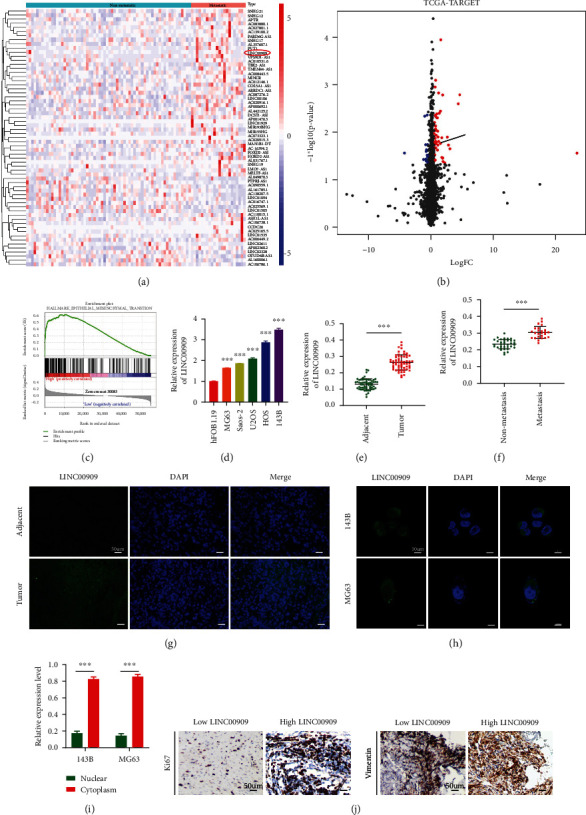
LINC00909 is highly expressed in OS tissues and cells. (a, b) The cluster heat map (a) and volcano plot (b) compared the differentially expressed lincRNAs between metastatic cases and nonmetastatic OS samples in TCGA-TARGET; (c) GSEA was used to compare the high LINC00909 group (red) with the low LINC00909 group (blue) among the OS cohorts in TCGA-TARGET dataset; (d) RT-qPCR detected LINC00909 expression in OS cell lines and hFOB1.19 (*n* = 3); (e) RT-qPCR detected LINC00909 expression in 60 pairs of clinical OS specimens and matched adjacent normal tissues; (f) the expression level of LINC00909 in patients with and without pulmonary metastasis; (g) FISH was performed to measure the expression of LINC00909 in clinical tumor and adjacent samples (*n* = 4); (h, i) the majority of LINC00909 was located in the cytoplasm according to FISH (h) and the nuclear mass separation assay (*n* = 4) (i). (j) Immunohistochemical staining of ki-67 and vimentin in selected clinical samples of the same T2N1M1 stage (*n* = 4). Data are presented as the means ± SD. ^∗^*p* < 0.05, ^∗∗^*p* < 0.01, and ^∗∗∗^*p* < 0.001.

To identify potential lncRNAs which participate in OS metastasis, we first explored the TARGET database and found that lncRNA LINC00909 was highly expressed in OS metastatic specimens compared with nonmetastatic samples (Figures 1(a) and 1(b)). Moreover, the results of GSEA indicated that LINC00909 may facilitate the EMT process in OS (Figure 1(c)). We therefore chose LINC00909 for further study. LINC00909 expression in OS cells and HFOB1.19 was detected through RT-qPCR. The expression level of LINC00909 was higher in OS cells compared with healthy hFOB 1.19 cells (Figure 1(d)). Additionally, LINC00909 levels were measured in 60 OS tissues and matched noncarcinoma tissues. LINC00909 expression levels were higher in OS samples compared with matched noncarcinoma samples (Figure 1(e)). At last, in comparison with the nonmetastatic group, LINC00909 levels increased within the metastatic group (Figure 1(f)). Moreover, we further verified that LINC00909 was highly expressed in OS samples compared with matched noncarcinoma samples by FISH (Figure 1(g)). We also performed nuclear mass separation and FISH assays to determine the subcellular localization of LINC00909 in OS cells; LINC00909 was found mainly in the cytoplasm (Figures 1(h) and 1(i)). IHC analysis of clinical samples revealed that Ki-67 and vimentin expression in the high-LINC00909 group was significantly higher relative to that in the low-LINC00909 group (Figure 1(j)). In addition, we examined the association of LINC00909 levels with clinicopathological characteristics in the 60 OS cases (Table 1), LINC00909 expression was in direct proportion to tumor size, metastasis, and TNM stage. Taken together, LINC00909 is highly expressed in OS tissues and cells and may be associated with patient prognosis.

### 3.2. Overexpressing LINC00909 Promotes Proliferation, Migration, Invasion, and EMT of OS Cells *In Vitro*

**Figure 2:**
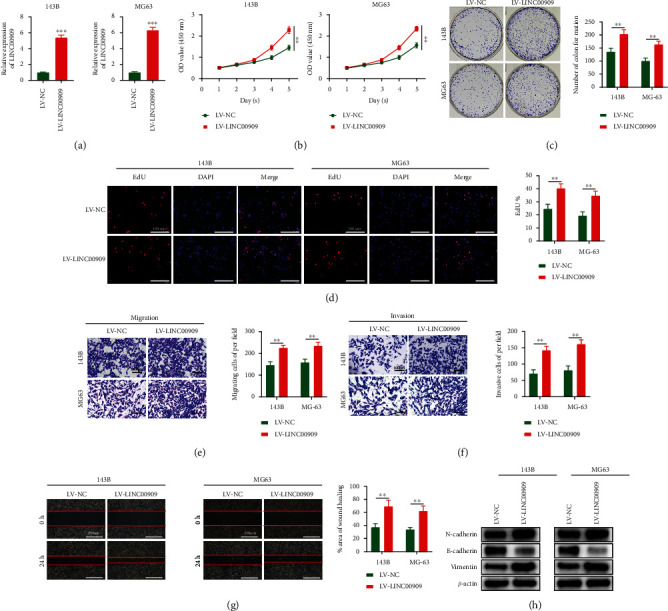
Overexpressing LINC00909 promotes proliferation, migration, invasion, and EMT of OS cells *in vitro*. (a) The expression of LINC00909 in 143B and MG63 cells transfected with LV-LINC00909 (*n* = 3); (b–d) CCK-8 (b), colony formation (c), and EdU assays (d) were used to detect the effect of LINC00909 on the proliferation of OS cells in *vitro* (*n* = 4); (e–g) transwell migration (e), transwell invasion (f), and scratch assays (g) were used to evaluate the effect of LINC00909 on OS cell migration and invasion (*n* = 4); (h) western blot analysis of EMT-related proteins following LV-LINC00909 (*n* = 3). Data are presented as the means ± SD. ^∗^*p* < 0.05, ^∗∗^*p* < 0.01, and ^∗∗∗^*p* < 0.001.

We investigated the effect of overexpressing LINC00909 on proliferation, migration, invasion, and EMT in OS cells. LV-LINC00909 was transfected into MG63 and 143B cells. RT-qPCR was conducted to assess gene expression posttransfection (Figure 2(a)). The CCK-8 assay data suggested that the LV-LINC00909 group had significantly higher cell proliferation (Figure 2(b)). In addition, OS cells showed enhanced colony formation abilities in the LV-LINC00909 group (Figure 2(c)). Furthermore, based on the EdU assay, LINC00909 overexpression resulted in an increase in the mitotic cell proportion in the LV-LINC00909 group (Figure 2(d)). Scratch and transwell assays were performed to investigate the function of LINC00909 in OS cell migration and invasion. LV-LINC00909 overexpression increased the proportion of migrating cells and promoted cell invasion (Figures 2(e) and 2(f)). The LV-LINC00909 group showed an elevated migration rate in the scratch assay (Figure 2(g)). Subsequently, we examined the association between LINC00909 and EMT-associated proteins through WB. Vimentin and N-cadherin levels were upregulated after LINC00909 overexpression, while E-cadherin levels were attenuated (Figure 2(h)), suggesting that LINC00909 activates EMT to enhance tumor metastasis. Taken together, in vitro experiments demonstrate that LINC00909 stimulates OS cell growth, migration, invasion, and EMT.

### 3.3. Downregulation of LINC00909 Inhibits OS Cell Proliferation, Migration, Invasion, and EMT *In Vitro*

**Figure 3:**
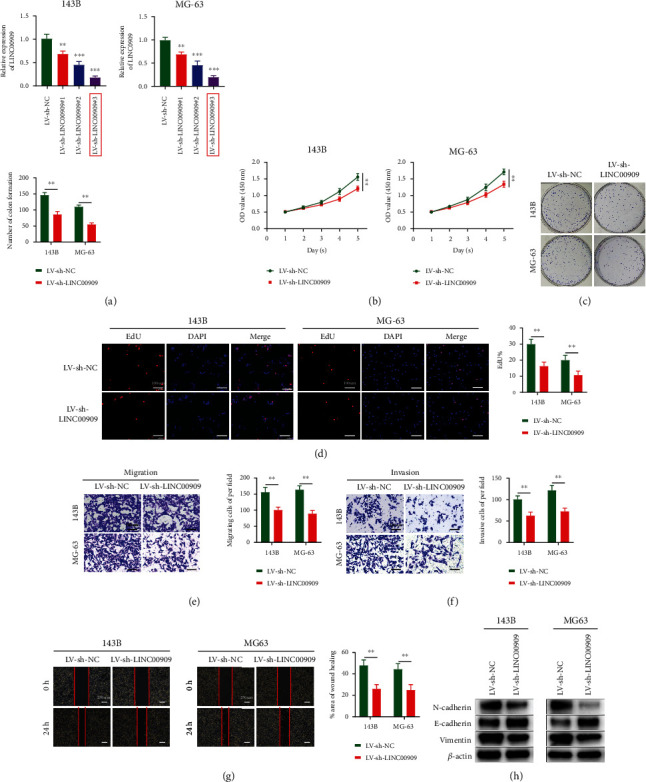
Downregulation of LINC00909 inhibits OS cell proliferation, migration, invasion, and EMT *in vitro*. (a) The expression of LINC00909 in 143B and MG63 cells transfected with LV-sh-LINC00909 (*n* = 3); (b–d) CCK-8 (b), colony formation (c), and EdU assays (d) were used to detect the effect of LV-sh-LINC00909 on the proliferation of OS cells in *vitro* (*n* = 4); (e–g) transwell migration (e), transwell invasion (f), and scratch assays (g) were used to evaluate the effect of LV-sh-LINC00909 on OS cell migration and invasion (*n* = 4); (h) western blot analysis of EMT-related proteins following LV-sh-LINC00909 (*n* = 3). Data are presented as the means ± SD. ^∗^*p* < 0.05, ^∗∗^*p* < 0.01, and ^∗∗∗^*p* < 0.001.

We next investigated whether LINC00909 knockdown affected the proliferation, migration, invasion, and EMT of OS cells. 143B and MG63 cell lines were transfected with LV-sh-LINC00909 (Figure 3(a)). We chose LV-sh-LINC00909#3 for shRNA experiments, since it had the greatest knockdown activity. To investigate whether LINC00909 exerts a critical role in OS cell proliferation, we performed CCK-8, colon formation, and EdU assays after LINC00909 downregulation. Cell proliferation, colon formation abilities, and the proportion of mitotic cells were all decreased after LINC00909 downregulation (Figures 3(b)–3(d)). We also investigated the effect of LINC00909 knockdown on the invasion and migration of OS cells using scratch and transwell assays. Migrating cell numbers decreased with LINC00909 knockdown (Figure 3(e)). The low expression level of LINC00909 also resulted in a reduced invasive capacity of OS cells (Figure 3(f)). Furthermore, the LV-sh-LINC00909 group had a decreased migration rate (Figure 3(g)). Vimentin and N-cadherin expression was also decreased after LINC00909 downregulation, whereas E-cadherin expression increased (Figure 3(h)). In summary, the in *vitro* data suggest that LINC00909 inhibition suppresses proliferation, migration, invasion, and EMT of OS cells.

### 3.4. LINC00909 Promotes OS Tumorigenesis and Metastasis *In Vivo*

**Figure 4:**
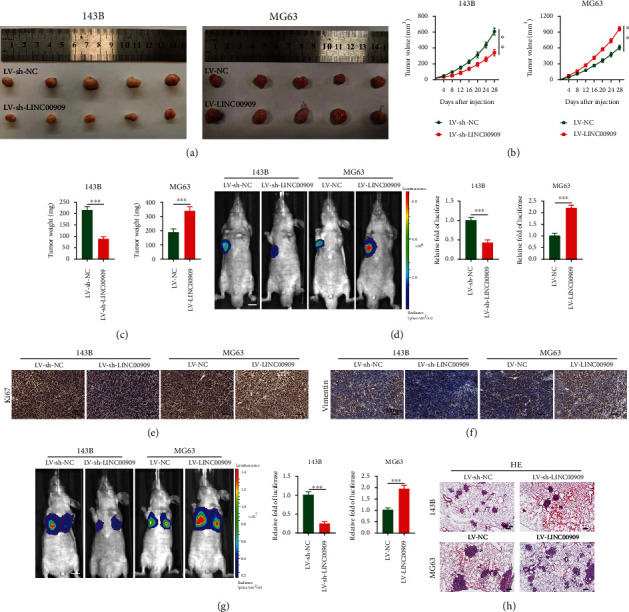
LINC00909 promotes OS tumorigenesis and metastasis *in vivo*. (a) Images of tumors obtained from mice treated with LV-LINC00909 and LV-sh-LINC00909 and their negative controls *n* = 5 mice/group; (b, c) tumor volume (b) and weight (c) were calculated (*n* = 5); (d) representative images of tumors were obtained by the IVIS imaging system (*n* = 5); (e, f) immunohistochemical analysis was performed to evaluate ki-67 (e) and vimentin (f) expression in *vivo* (*n* = 5); (g) representative images of pulmonary metastases were obtained by the IVIS imaging system (*n* = 5); (h) representative H&E-stained lung sections from mice in different groups (*n* = 5). Data are presented as the means ± SD. ^∗^*p* < 0.05, ^∗∗^*p* < 0.01, and ^∗∗∗^*p* < 0.001.

We conducted a mouse model to further investigate the role of LINC00909 in OS tumorigenesis in *vivo*. We constructed a tumor model in nude mice using subcutaneous injection of fluorescein-expressing stably transfected OS cells. After four weeks, the LV-LINC00909 group had elevated tumor weight and volume relative to the LV-NC group, whereas the LV-sh-LINC00909 group had reduced tumor weight and volume relative to the LV-sh-NC group (Figures 4(a)–4(c)). The LV-LINC00909 group had a significantly increased tumor volume compared with the LV-NC group, whereas the LV-sh-LINC00909 group had a decreased tumor volume compared with the LV-sh-NC group, as shown in the in vivo imaging experiments (Figure 4(d)). As revealed by tissue IHC, vimentin and Ki-67 levels were elevated in the LV-LINC00909 group, while vimentin and Ki-67 levels were decreased in the LV-sh-LINC00909 group (Figures 4(e) and 4(f)). We also constructed an in *vivo* nude mouse model of OS lung metastasis for elucidating the effects of LINC00909 on OS metastasis. We assigned animals into four groups: LV-LINC00909, LV-sh-LINC00909, and the corresponding NC groups. The LV-LINC00909 group had remarkably enhanced lung metastases, whereas the LV-sh-LINC00909 group had decreased lung metastases. These observations were confirmed through in *vivo* imaging (Figure 4(g)). Lung metastatic lesions were verified through hematoxylin and eosin staining (Figure 4(h)). Altogether, our results show that LINC00909 promotes OS tumorigenesis and metastasis in *vivo*.

### 3.5. LINC00909 Functions as a Molecular Sponge for miR-875-5p

**Figure 5:**
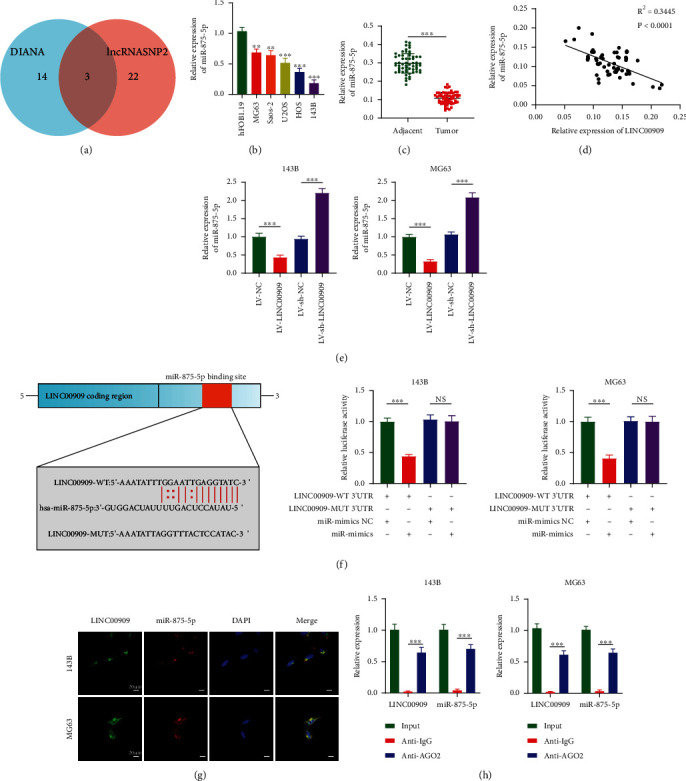
LINC00909 functions as a molecular sponge for miR-875-5p. (a) Bioinformatics analysis showed that LINC00909 has a total of 3 target miRNAs in DIANA and lncRNASNP2. (b, c) The expression level of miR-875-5p in OS cell lines and hFOB1.19 (*n* = 3) (b) and clinical samples (c). (d) Linear regression analysis showed that LINC00909 was negatively correlated with the expression of miR-875-5p in OS tissues. (e) The expression of miR-875-5p in OS cells after alteration of LINC00909 expression was detected by RT-qPCR (*n* = 3). (f) Luciferase reporter assay was performed to confirm that miR-875-5p directly bound to the 3′-UTR region of LINC00909. Luciferase activity was analyzed in OS cells cotransfected with miR-875-5p mimics or negative control with pGL3- LINC00909-WT or pGL3-LINC00909-MUT (*n* = 5). (g) The FISH assay revealed the colocalization of LINC00909 with miR-875-5p (*n* = 3). (h) RIP assay was conducted to examine miR-875-5p endogenously associated with LINC00909 (*n* = 3). Data are presented as the means ± SD. ^∗^*p* < 0.05, ^∗∗^*p* < 0.01, and ^∗∗∗^*p* < 0.001.

lincRNAs can bind various miRNAs to suppress miRNA expression and reduce their regulation of target mRNAs. By using the online databases, DIANA and lncRNASNP2, we predicted three potential target miRNAs with high binding scores (Figure 5(a)). Among the three candidate target miRNAs, we selected miR-875-5p for subsequent experiments because it participates in different cancers and has an important function in suppressing cancer development. miR-875-5p levels were measured in hFOB1.19 osteoblasts and five types of OS cells using RT-qPCR. And miR-875-5p levels within OS cells remarkably decreased compared with hFOB1.19 cells (Figure 5(b)). RT-qPCR analysis also demonstrated downregulation of miR-875-5p in OS tissues compared with matched noncarcinoma tissues (Figure 5(c)). In addition, miR-875-5p showed a negative correlation with LINC00909 in OS clinical samples (Figure 5(d)). Based on the endogenous miR-875-5p expression levels in OS cells, 143B and MG63 cell lines were transfected with miR-875-5p mimics or miR-875-5p inhibitor, respectively. RT-qPCR was later conducted to assess transfection efficiency (Additional file 3: Figure S1(a)). To investigate the function of miR-875-5p in the proliferation of OS cells, we performed CCK-8 and EdU assays after miR-875-5p upregulation. The results demonstrate that cell proliferation was inhibited after miR-875-5p upregulation (Additional file 3: Figures S1(b) and S1(c)). We also explored the effect of miR-875-5p on the migration and invasion of OS cells using transwell assays. The number of migrating cells decreased (Additional file 3: Figure S1(d)), and the invasive capacity of OS cells was reduced (Additional file 3: Figure S1(e)) when miR-875-5p was upregulated. Furthermore, we observed that LINC00909 overexpression downregulated miR-875-5p expression levels, and LV-sh-LINC00909 upregulated miR-875-5p expression levels in 143B and MG63 cells (Figure 5(e)). Moreover, the dual-luciferase reporter assay showed that miR-875-5p overexpression dramatically decreased WT LINC00909 luciferase activity but had little effect on MUT LINC00909 (Figure 5(f)). FISH analysis indicated that LINC00909 was bound to miR-875-5p (Figure 5(g)). RIP assay showed that miR-875-5p and LINC00909 were enriched in Ago2 immunoprecipitants compared with the control IgG immunoprecipitant (Figure 5(h)). These data implied that LINC00909 could function as a molecular sponge for miR-875-5p.

### 3.6. HOXD9 Is Upregulated and Is a Target Gene of miR-875-5p in OS

**Figure 6:**
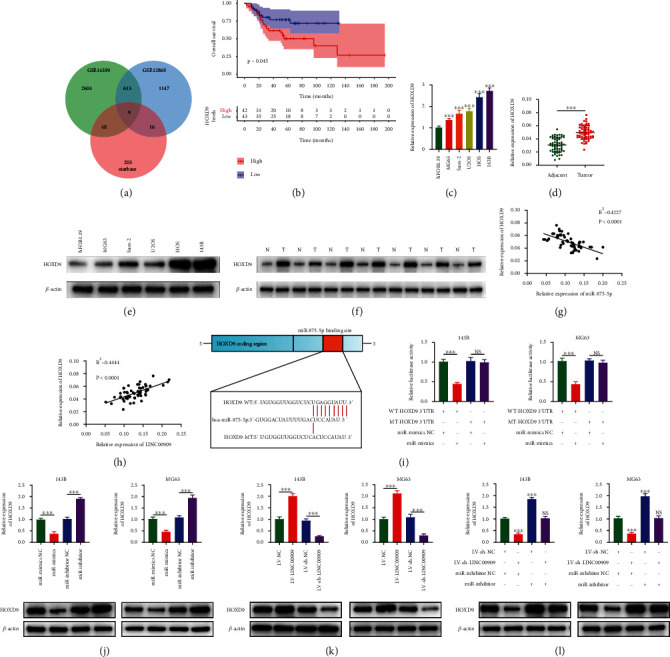
HOXD9 is upregulated and is a target gene of miR-875-5p in OS. (a) Bioinformatics analysis showed that miR-875-5p has a total of 9 target genes in starBase, GSE14359, and GSE12865. (b) Kaplan-Meier analysis demonstrated that patients with low HOXD9 expression levels had a better prognosis according to the TARGET database. (c, d) The mRNA expression level of HOXD9 in OS cell lines and hFOB1.19 (*n* = 3) (c) and clinical samples (d). (e, f) The protein level of HOXD9 in OS cell lines and hFOB1.19 (*n* = 3) (e) and clinical samples (N means normal tissues and T stands for tumor) (f). (g) Linear regression analysis showed that HOXD9 was negatively correlated with the expression of miR-875-5p in OS tissues. (h) Positive correlation between HOXD9 and LINC00909 expression in OS tissues. (i) Luciferase reporter assay was performed to confirm that miR-875-5p directly bound to the 3′-UTR region of HOXD9. Luciferase activity was analyzed in OS cells cotransfected with miR-875-5p mimics or negative control with pGL3-HOXD9-WT or pGL3-HOXD9-MUT (*n* = 5). (j–l) The expression of HOXD9 in OS cells after alteration of miR-875-5p and LINC00909 expression was detected by RT-qPCR and WB (*n* = 3). Data are presented as the means ± SD. ^∗^*p* < 0.05, ^∗∗^*p* < 0.01, and ^∗∗∗^*p* < 0.001.

To find miR-875-5p's downstream targets, the starBase online tool was used and then overlapped with DEGs from GSE14359 and GSE12865, which resulted in nine shared genes (Figure 6(a)). We then adopted a Kaplan–Meier approach for exploring the association of overall survival with these nine genes in patients based on the TARGET database (Additional file 4: Figure S2). As suggested by the Kaplan–Meier survival method, HOXD9 upregulation predicted poor overall survival for OS cases (*p* < 0.05) (Figure 6(b)). Therefore, we predicted HOXD9 as a putative target for miR-875-5p. As a follow-up, we conducted WB and RT-qPCR on 60 clinical sample pairs and six cell samples. As a result, HOXD9 was dramatically upregulated in OS cells and tissues (Figures 6(c)–6(f)). Moreover, HOXD9 showed a negative correlation with miR-875-5p but a positive correlation with LINC00909 in OS clinical samples (Figures 6(g) and 6(h)). miR-875-5p overexpression reduced WT HOXD9 luciferase activity but made no significant difference to MUT HOXD9, as evidenced by the dual-luciferase reporter assay (Figure 6(i)). Additionally, miR-875-5p overexpression downregulated HOXD9, while the miR-875-5p inhibitor upregulated HOXD9 expression levels in OS cells, as assessed by RT-qPCR and WB assays (Figure 6(j)). Moreover, LINC00909 overexpression upregulated HOXD9 expression levels while LINC00909 downregulation reduced HOXD9 expression levels (Figure 6(k)). In the rescue experiment, LINC00909 downregulation eliminated miR-875-5p inhibitor-induced HOXD9 upregulation (Figure 6(l)). These data implied that HOXD9 is upregulated and is a target gene of miR-875-5p in OS.

### 3.7. Downregulation of HOXD9 Inhibits OS Cell Proliferation, Migration, Invasion, and EMT In Vitro

**Figure 7:**
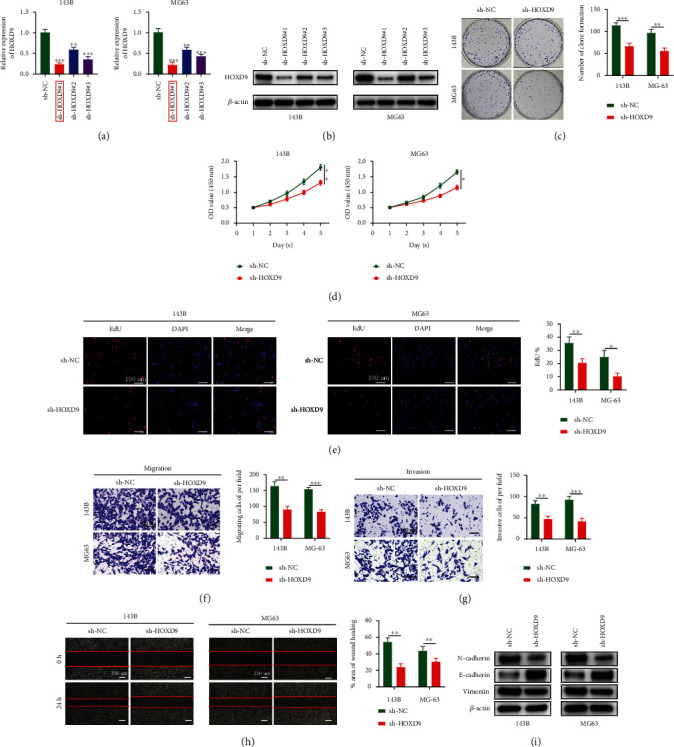
Downregulation of HOXD9 inhibits OS cell proliferation, migration, invasion, and EMT in *vitro*. (a, b) The expression of HOXD9 in 143B and MG63 cells transfected with sh-HOXD9 was detected by RT-qPCR (a) and WB (*n* = 3) (b). (c–e) Colony formation (c), CCK-8 (d), and EdU (e) assays were used to detect the effect of sh-HOXD9 on OS cell proliferation in vitro (*n* = 4). (f–h) Transwell migration (f), transwell invasion (g), and scratch assays (h) were used to evaluate the effect of sh-HOXD9 on cell migration and invasion (*n* = 4). (i) Western blot analysis of EMT-related proteins following sh-HOXD9 (*n* = 3). Data are presented as the means ± SD. ^∗^*p* < 0.05, ^∗∗^*p* < 0.01, and ^∗∗∗^*p* < 0.001.

Based on endogenous HOXD9 expression level in OS cells, we transfected sh-HOXD9 plasmid into MG63 and 143B cells. The expression level after transfection was assessed by RT-qPCR and WB (Figures 7(a) and 7(b)). We chose sh-HOXD9#1 for shRNA experiments, since it had the greatest suppression activity. For exploring the function of HOXD9 during OS cell proliferation, we performed CCK-8, colony formation, and EdU assays after HXOD9 knockdown. The colony forming ability of OS cells was decreased after HOXD9 downregulation (Figure 7(c)). CCK-8 assay revealed remarkably inhibited cell proliferation following HOXD9 downregulation (Figure 7(d)). In addition, the EdU proliferation assay showed that the mitotic cell proportion declined following decreased HOXD9 expression (Figure 7(e)). We assessed the role of HOXD9 in the migration and invasion of OS cells using scratch and transwell assays. The migrating cell number decreased when HOXD9 was knocked down (Figure 7(f)). The ability of cell migration and invasion decreased in the sh-HOXD9 group (Figures 7(g) and 7(h)). Furthermore, N-cadherin and vimentin levels were attenuated after HOXD9 downregulation, while E-cadherin was upregulated (Figure 7(i)), suggesting that HOXD9 activates EMT to enhance tumor metastasis. Taken together, the in vitro experiments showed that downregulation of HOXD9 inhibited OS cell proliferation, migration, invasion, and EMT.

### 3.8. LINC00909 Regulates the Expression of HOXD9 by Acting as a ceRNA of miR-875-5p to Promote OS Cell Proliferation, Migration, Invasion, and EMT

**Figure 8:**
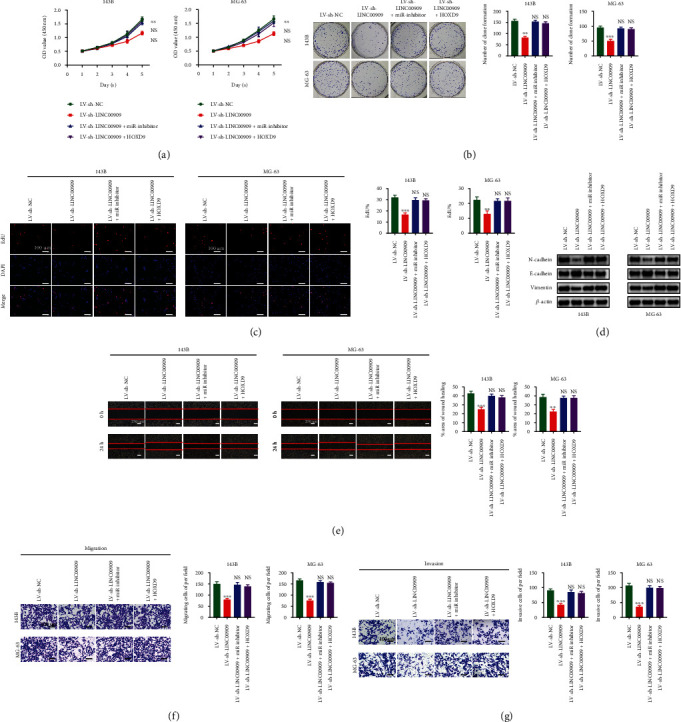
LINC00909 regulates the expression of HOXD9 by acting as a ceRNA of miR-875-5p to promote OS cell proliferation, migration, invasion, and EMT. Rescue experiments for LV-sh-LINC00909 were conducted by downregulating miR-875-5p or upregulating HOXD9 in OS cells. (a–c) Colony formation (a), CCK-8 (b), and EdU assays (c) were conducted to detect the proliferation ability of OS cells (*n* = 4). (d) Western blot analysis was conducted to evaluate the EMT-related proteins level (*n* = 3). (e–g) Rescue experiments were also conducted using the scratch (e), transwell migration (f), and transwell invasion assays (g) (*n* = 4). Data are presented as the means ± SD. ^∗^*p* < 0.05, ^∗∗^*p* < 0.01, and ^∗∗∗^*p* < 0.001.

We next sought to confirm the role of LINC00909 as a ceRNA that binds miR-875-5p and regulates HOXD9 expression to promote the proliferation, migration, invasion, and EMT of OS cells by a series of rescue experiments. CCK-8, colony formation, and EdU assays showed that cell proliferation was significantly inhibited with LINC00909 downregulation, while overexpression of HOXD9 or downregulation of miR-875-5p could reverse this effect (Figures 8(a)–8(c)). WB showed that LINC00909 downregulation attenuated expression levels of N-cadherin and vimentin, while E-cadherin levels were upregulated, and HOXD9 overexpression or downregulation of miR-875-5p could reverse this effect (Figure 8(d)). Scratch and transwell assays demonstrated that overexpression of HOXD9 or downregulation of miR-875-5p reversed the suppressive impact of LV-sh-LINC00909 on invasion and migration of OS cells (Figures 8(e)–8(g)). In conclusion, LINC00909 promotes OS cell proliferation, migration, invasion, and EMT by acting as a ceRNA to bind miR-875-5p while modulating HOXD9.

### 3.9. The LINC00909/miR-875-5p/HOXD9 Axis Regulates the PI3K/AKT/mTOR Signaling Pathway

**Figure 9:**
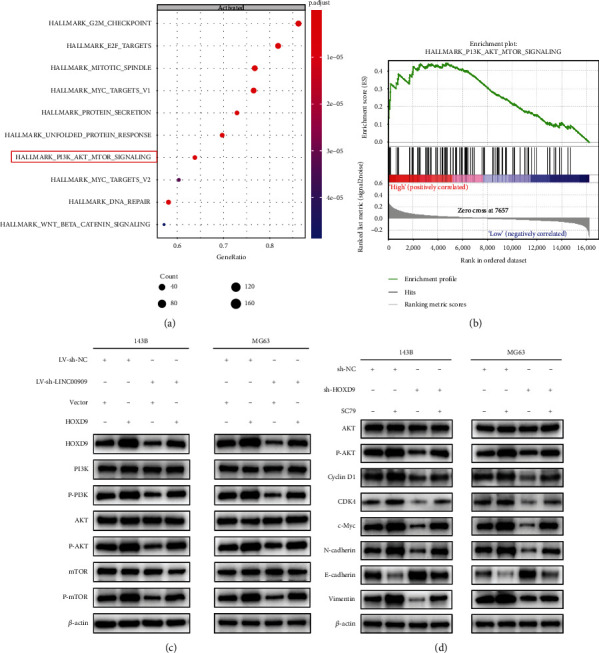
The LINC00909/miR-875-5p/HOXD9 axis regulates the PI3K/AKT/mTOR signaling pathway. (a, b) Single-gene GSEA showed top activated gene sets ordered by gene ratio (a), and the PI3K/AKT/mTOR signaling pathway was enriched after HOXD9 overexpression (b). (c) Representative images of western blot analysis of the levels of HOXD9, P-PI3K, PI3K, P-AKT, AKT, P-mTOR, and mTOR in transfected OS cells (*n* = 3). (d) Representative images of western blot analysis of P-AKT, AKT, N-cadherin, vimentin, E-cadherin, c-Myc, CDK4, and cyclin D1 levels in transfected MG63 and 143B cells treated with the AKT activator SC79 (*n* = 3).

To explore the downstream mechanism of the LINC00909/miR-875-5p/HOXD9 axis, we performed GSEA to measure the downstream signaling pathway of HOXD9 in OS cells. Enrichment of the PI3K/AKT/mTOR pathway was observed when HOXD9 was highly expressed (Figures 9(a) and 9(b)). As reported previously, the PI3K/AKT/mTOR pathway is involved in tumor progression. We next investigated whether the LINC00909/miR-875-5p/HOXD9 axis promotes OS progression through the PI3K/AKT/mTOR pathway. Upregulating HOXD9 increased protein levels of HOXD9, P-PI3K, P-AKT, and P-mTOR, but downregulating LINC00909 reversed these effects (Figure 9(c)). Moreover, treating OS cells with AKT agonist (SC79) reversed the inhibitory effect of sh-HOXD9 on the expression of the G1/S checkpoint proteins and EMT-related proteins (Figure 9(d)). Overall, the above findings verify the involvement of the LINC00909/miR-875-5p/HOXD9 axis in regulating OS malignant progression via the PI3K/AKT/mTOR pathway (Additional file 5: Figure S3).

## 4. Discussion

The literature has shown that lncRNAs play an extremely significant role in the malignant progression of OS. For example, the lncRNA lncARSR promotes OS progression by activating AKT [26]. lncRNA SNHG4 facilitates OS progression by binding miR-224-3p [27]. LINC00909 is highly expressed in a variety of tumors and is closely related to a poor prognosis. For instance, LINC00909 is highly expressed in ovarian cancer and significantly enhances the proliferation and metastasis of ovarian cancer cells [10]. However, the role of LINC00909 in OS has yet to be explored. In our study, we found that LINC00909 was highly expressed in OS metastatic cases in the TARGET database and positively correlated with EMT-associated gene signatures through bioinformatics analysis and RT-qPCR shows that LINC00909 levels are upregulated in OS cells and tissues. Moreover, LINC00909 expression showed a positive correlation with tumor size, ALP, metastasis, and TNM stage. According to experiments in *vivo* and in *vitro*, LINC00909 enhances the proliferation, migration, invasion, and EMT of OS cells. Therefore, we conclude that LINC00909 can serve as an oncogenic lncRNA in OS.

Previous studies have found that miRNAs play an important role in the pathogenesis of tumors [28–30]. Furthermore, miRNAs play a vital role in the occurrence and development of OS [31, 32]. Although miR-875-5p is involved in inhibiting tumor progression in a variety of tumors [18, 19], the mechanism of action of miR-875-5p in OS has not previously been studied. We found that miR-875-5p expression was markedly decreased in OS cells and tissues. *In vitro* experiments also showed that the proliferation, migration, and invasion ability of OS was significantly reduced after miR-875-5p overexpression. Moreover, FISH, RIP, and luciferase assays demonstrated that miR-875-5p was a downstream target of LINC00909. These data suggest that LINC00909 exerts oncogenic effects by binding to miR-875-5p in OS.

Recent studies have demonstrated that lncRNAs can play the role of ceRNAs that bind to miRNAs, which are involved in regulating target genes, thus affecting the development of tumors, including OS [33, 34]. For example, *in vivo* and *in vitro* experimental results show that lncRNA NEAT1 significantly promotes OS progression through the ceRNA mechanism [33]. Therefore, whether LINC00909 regulates the malignant progression of OS through the ceRNA mechanism should be explored.

HOXD9 is one of the homeobox family members, which has a critical function in the morphogenesis of multicellular organisms [35]. Moreover, the expression of HOXD9 increases in diverse cancers and is correlated with patient prognosis [36]. Through a bioinformatics analysis, we suggest that HOXD9 is a potential target of miR-875-5p. HOXD9 is significantly upregulated in OS cells and tissues and is negatively correlated with miR-875-5p expression. Dual-luciferase reporter assay confirmed that HOXD9 is a downstream target of miR-875-5p. Rescue experiments also confirmed that LINC00909 promotes OS cell proliferation, migration, invasion, and EMT by playing the role of the ceRNA to bind to miR-875-5p and regulate HOXD9.

The PI3K/AKT/mTOR pathway has been suggested to be involved in tumorigenesis [37, 38]. Moreover, the occurrence and development of OS are closely related to the signaling pathway [39]. Through a bioinformatics analysis of HOXD9 expression and the verification of proteins associated with the pathway by WB, we found that the LINC00909/miR-875-5p/HOXD9 axis regulates OS cell proliferation and EMT through the PI3K/AKT/mTOR pathway.

## 5. Conclusion

This work was conducted first to identify whether LINC00909 induces EMT and contributes to OS tumorigenesis and metastasis via the PI3K/AKT/mTOR pathway by binding to miR-875-5p to elevate HOXD9 expression. The present work illustrates the possible OS development mechanism and provides a novel anti-OS therapeutic strategy. Nonetheless, the OS pathogenic mechanism remains to be further elucidated.

## Figures and Tables

**Table 1:** Expression of LINC00909 according to patients' clinical features.

Characteristics		LINC00909 expression		*p* value
	Number	High group	Low group	
Age (y)				
<18	34	16	18	0.602
≥18	26	14	12	
Gender				
Female	28	15	13	0.605
Male	32	15	17	
Location				
Femur/tibia	27	10	17	0.069
Elsewhere	33	20	13	
TNM stage				
I	33	11	22	0.004^a^
II/III	27	19	8	
Tumor size (cm)				
<5	34	12	22	0.009^a^
≥5	26	18	8	
Lung metastasis				
Yes	25	17	8	0.018^a^
No	35	13	22	

^a^ *P* < 0.05 (chi-square test).

## Data Availability

Most of the datasets supporting the conclusions of this article are included within this article and the additional files. The datasets used or analyzed during the current study are available on reasonable request.

## Supplementary Materials

Supplementary Materials Additional file 1: Table S1: sequences used in this study. Additional file 2: Table S2: details of primary antibodies applied in this study. Additional file 3: Figure S1: miR-875-5p inhibits OS cell proliferation, migration, and invasion *in vitro*. Additional file 4: Figure S2: Kaplan-Meier analysis of target genes. Additional file 5: Figure S3: graphic abstract of the LINC00909/miR-875-5p/HOXD9 axis.
