# Supplementary material for: Long Noncoding RNA LINC00909 Induces Epithelial-Mesenchymal Transition and Contributes to Osteosarcoma Tumorigenesis and Metastasis
Source: J Oncol. 2022 Oct 10;2022:8660965. doi: 10.1155/2022/8660965 (PMC9576421; doi:10.1155/2022/8660965)

**Supplementary Information**

**Additional file 1: TABLE S1.** Sequences used in this study.

| **ID** | **Sequences** **(5′ →3′)** |
| --- | --- |
| LINC00909 forward | CAGCAAGCATGCCTTCACATC |
| LINC00909 reverse | CAAGTGCCACAGGTAGAGCT |
| hsa-miR-875-5p forward | GGCGCGTATACCTCAGTTTTATCA |
| hsa-miR-875-5p reverse | GTCGTATCCAGTGCAGGGTC |
| HOXD9 forward | CTCAGCAACTACTACGTGGACT |
| HOXD9 reverse | CAAAACTACACGAGGCGAACT |
| β-actin forward | GTGCACCTGACTCCTGAGGA |
| β-actin reverse | CTTGATACCAACCTGCCCAG |
| U6 forward | CTCGCTTCGGCAGCACA |
| U6 reverse | AACGCTTCACGAATTTGCGT |

**Additional file 2: TABLE S2.** Details of primary antibodies applied in this study.

| **Gene specificity** | **Manufacture of primary antibody** | **Dilution rate**  **(WB)** | **Dilution rate**  **(IHC)** | **Specificity** | **Catalog number** |
| --- | --- | --- | --- | --- | --- |
| HOXD9 | Proteintech | 1：500 |  | Rabbit | 20560-1-AP |
| c-Myc | Proteintech | 1：1000 |  | Rabbit | 10828-1-AP |
| CDK4 | Proteintech | 1：1000 |  | Rabbit | 11026-1-AP |
| cyclin D1 | Proteintech | 1：1000 |  | Rabbit | 26939-1-AP |
| N-cadherin | CST | 1：1000 |  | Rabbit | 13116S |
| Vimentin | CST | 1：1000 | 1：200 | Rabbit | 5741S |
| E-cadherin | CST | 1：1000 |  | Rabbit | 3195S |
| phospho-PI3K | CST | 1：1000 |  | Rabbit | 17366S |
| PI3K | CST | 1：1000 |  | Rabbit | 4249T |
| phospho-AKT | CST | 1：1000 |  | Rabbit | 4060T |
| AKT | CST | 1：1000 |  | Rabbit | 4691T |
| phospho-mTOR | CST | 1：1000 |  | Rabbit | 5536T |
| mTOR | CST | 1：1000 |  | Rabbit | 2983T |
| Ki-67 | Abcam |  | 1：200 | Rabbit | ab16667 |
| β-actin | CST | 1：1000 |  | Rabbit | 4970T |

**Additional file 3: FIGURE S1.** miR-875-5p inhibits OS cell proliferation, migration and invasion *in vitro*.


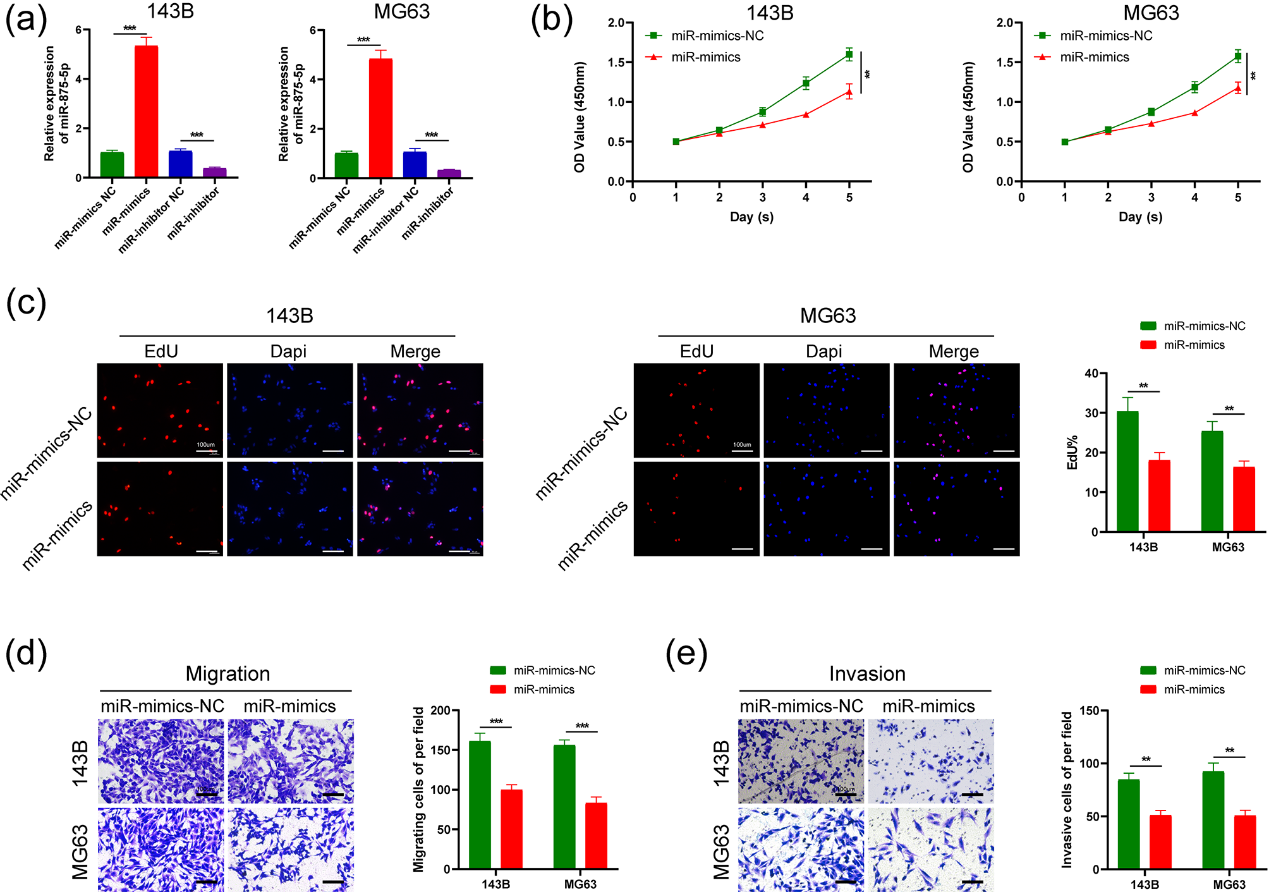


(**a**) The expression of miR-875-5p in OS cells transfected with miR mimics, miR inhibitor and their negative controls (n=3); (**b-c**) CCK-8 (**b**) and EdU (**c**) assays were used to detect the effect of miR-875-5p mimics on OS cells proliferation in *vitro* (n=4); (**d, e**) Transwell migration (**d**) and transwell invasion assays (**e**)were used to evaluate the effect of miR-875-5p mimics on OS cells migration and invasion (n=4); Data are presented as the means ± SD. **p* < 0.05, ***p* < 0.01, ****p* < 0.001.

**Additional file 4: FIGURE S2.** Kaplan-Meier analysis of target genes.


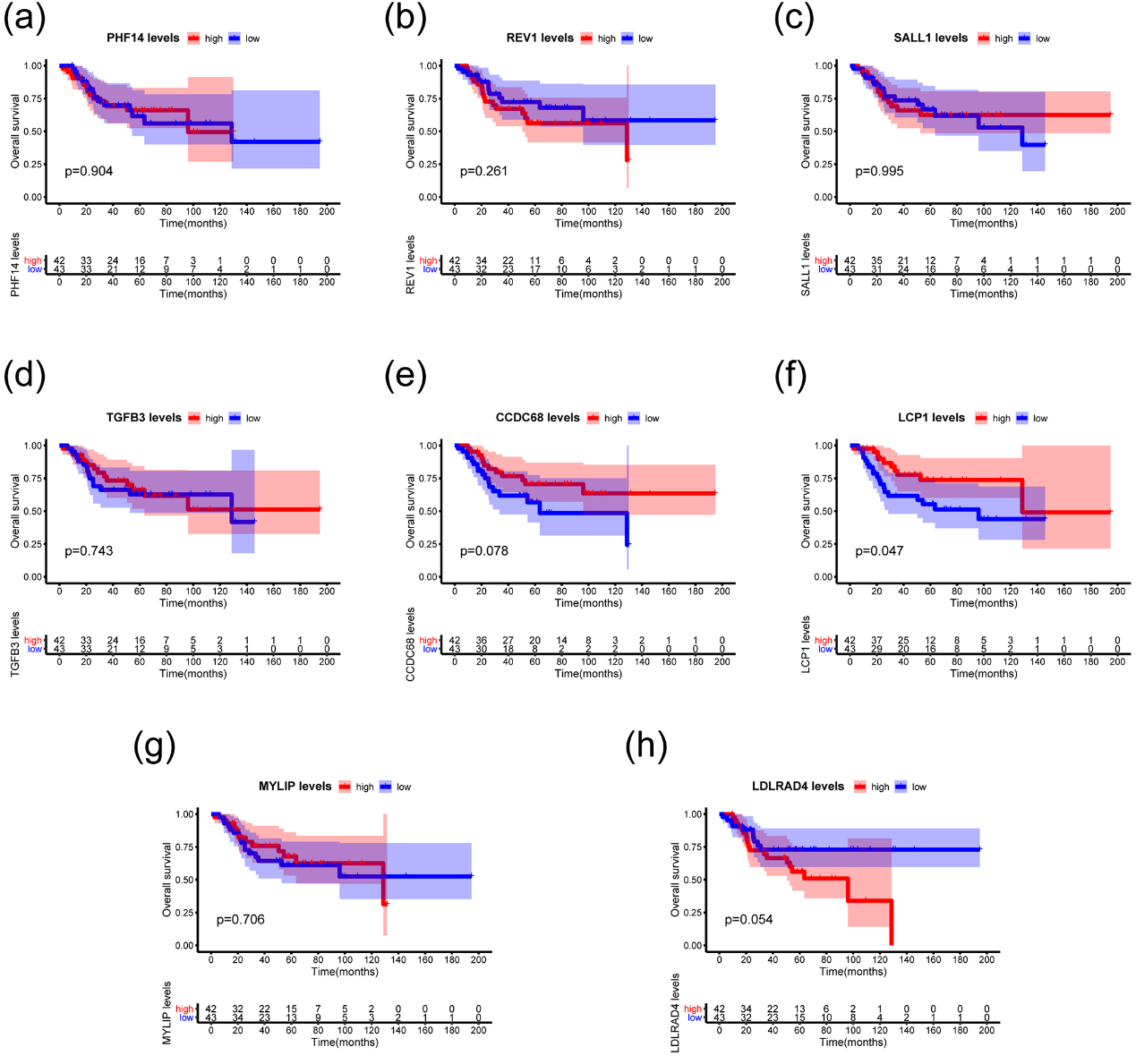


(**a-h**) Kaplan-Meier analysis of PHF14 (**a**), REV1 (**b**), SALL1 (**c**), TGFB3 (**d**), CCDC68 (**e**), LCP1 (**f**), MYLIP (**g**) and LDLRAD4 (**h**) according to TARGET database.

**Additional file 5: FIGURE S3.** Graphic abstract of the LINC00909/miR-875-5p/HOXD9 axis.


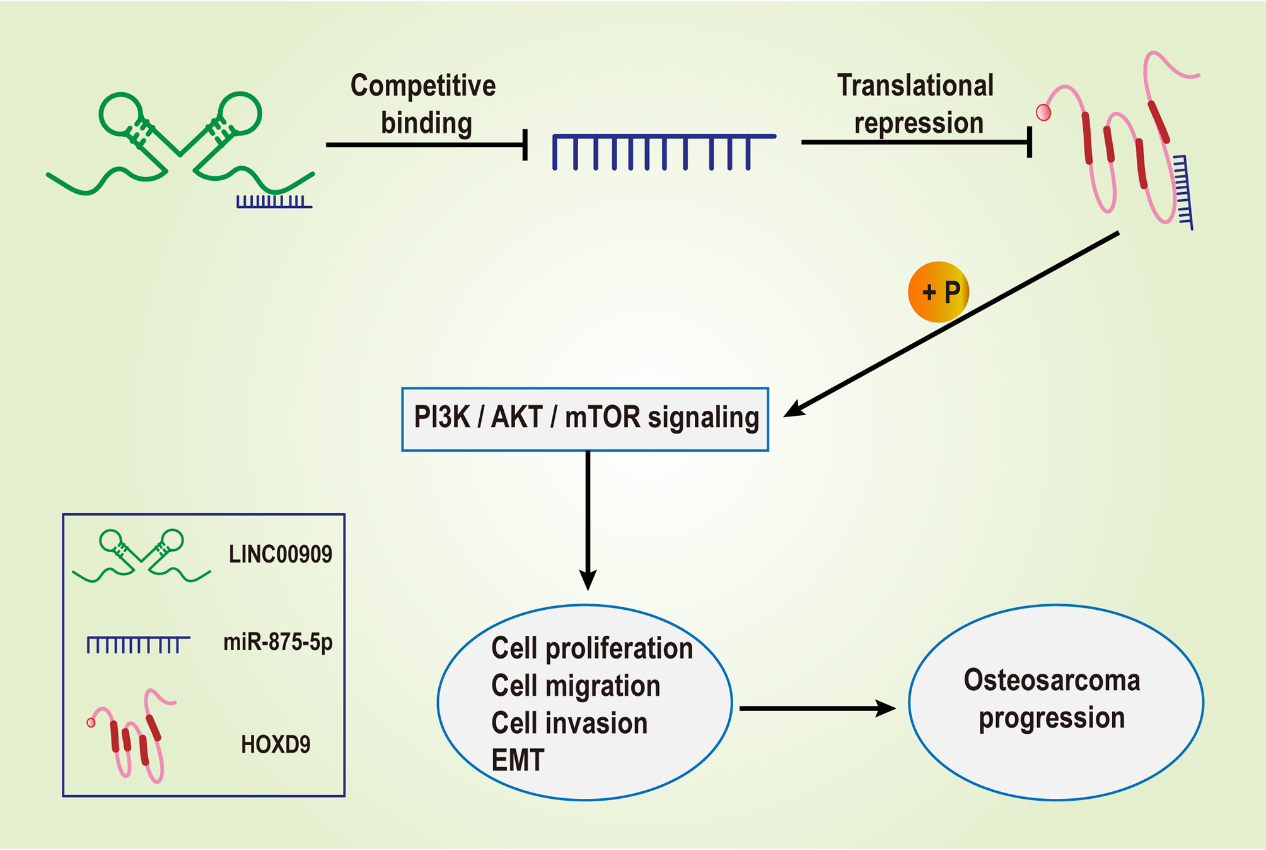

Supplement: Supplementary Materials — Additional file 1: Table S1: sequences used in this study. Additional file 2: Table S2: details of primary antibodies applied in this study. Additional file 3: Figure S1: miR-875-5p inhibits OS cell proliferation, migration, and invasion in vitro. Additional file 4: Figure S2: Kaplan-Meier analysis of target genes. Additional file 5: Figure S3: graphic abstract of the LINC00909/miR-875-5p/HOXD9 axis. [file 8660965.f1.docx]
